# Role of *Anopheles stephensi* Mosquitoes in Malaria Outbreak, Djibouti, 2019

**DOI:** 10.3201/eid2706.204557

**Published:** 2021-06

**Authors:** Vincent Pommier de Santi, Bouh Abdi Khaireh, Thomas Chiniard, Bruno Pradines, Nicolas Taudon, Sébastien Larréché, Abdoulraouf Bourhan Mohamed, Franck de Laval, Franck Berger, Florian Gala, Madjid Mokrane, Nicolas Benoit, Lionel Malan, Abdoulilah Ahmed Abdi, Sébastien Briolant

**Affiliations:** Aix Marseille University, IRD, AP-HM, SSA, VITROME, Marseille, France (V. Pommier de Santi, B. Pradines, N. Benoit, S. Briolant);; IHU-Méditerranée Infection, Marseille (V. Pommier de Santi, B. Pradines, N. Benoit, S. Briolant);; French Armed Forces Center for Epidemiology and Public Health, Marseille (V. Pommier de Santi, F. de Laval, F. Berger, M. Mokrane);; United Nations Development Program, The Global Fund to Fight AIDS, Tuberculosis and Malaria, Djibouti, Republic of Djibouti (B.A. Khaireh);; French Armed Forces Medical Health Service, Djibouti (T. Chiniard, F. Gala, L. Malan);; French Armed Forces Biomedical Research Institute, Marseille (B. Pradines, N. Taudon, N. Benoit, S. Briolant);; National Center for Malaria, Marseille (B. Pradines, N. Benoit);; Bégin Military Teaching Hospital, Paris, France (S. Larréché);; Djiboutian Gendarmerie Health Service, Djibouti (A. Bourhan Mohamed);; Aix Marseille University, INSERM, IRD, Sciences Economiques & Sociales de la Santé & Traitement de l’Information Médicale, Marseille (F. de Laval, F. Berger);; Djiboutian Armed Forces Health Service, Djibouti (A. Ahmed Abdi)

**Keywords:** malaria, *Anopheles stephensi*, urban malaria, outbreak, resistance, antimalarial drug, *Plasmodium falciparum*, *Plasmodium vivax*, Djibouti, mosquitoes, vector-borne infections, parasitic infections, mosquito-borne infections, parasites, antimicrobial resistance

## Abstract

*Anopheles stephensi* mosquitoes share urban breeding sites with *Aedes aegypti* and *Culex quinquefasciatus* mosquitoes in the Republic of Djibouti. We present evidence that *A. stephensi* mosquitoes might be responsible for an increase in malaria incidence in this country. We also document resistance of *Plasmodium falciparum* to dihydroartemisinin/piperaquine.

The Republic of Djibouti, bordered by Eritrea, Ethiopia, and Somalia, is a semiarid country in the Horn of Africa. The population comprises <900,000 persons, 70% of whom live in Djibouti, the capital city. Before 2013, malaria was hypoendemic to the country, with low levels of transmission in periruban and rural areas during December–May. Localized outbreaks occurred regularly, possibly caused by migration from surrounding countries. Most cases were caused by infection with *Plasmodium falciparum* (>80%) or *P. vivax*. Before 2013, researchers considered the *Anopheles arabiensis* mosquito to be the primary vector (*1*).

The incidence of malaria had drastically decreased in the country since 2008; by 2012, this transmission level was compatible with preelimination goals (*2*,*3*). In 2013, an autochthonous outbreak of malaria occurred in Djibouti; field entomologic investigations identified *An. stephensi* mosquitoes as a new malaria vector (*4*). This species, a known vector of urban malaria in India and the Arabian Peninsula, has changed the epidemiologic profile of malaria in Djibouti (*5*). In 2018, malaria incidence increased to 25,319 confirmed cases (64% caused by *P. falciparum* and 36% by *P. vivax*) and >100,000 suspected cases (Appendix Figure 1).

The French Armed Forces (FAF) have served in Djibouti for decades. Service members and their families (≈2,700 persons) live in the capital. Despite malaria prevention and treatment measures described elsewhere (*6*), an outbreak among French military personnel occurred in February 2019; failure of early artemisinin combined therapy was documented in 1 patient.

## The Study

We collated FAF epidemiologic surveillance data on malaria cases among service members in Djibouti during 1993–2019; the 2019 data included cases among family members. We defined a malaria case as an illness resulting in a positive result on a rapid diagnostic test or thin blood smear.

We conducted the field investigation in the capital during February 28–March 22, 2019. We obtained a dried blood spot on filter paper from each patient and stored the samples in a sealed plastic pouch until processing. We extracted DNA from the samples and confirmed diagnosis using PCR. We sequenced the antimalarial drug resistance molecular markers *Pfdhfr*, *Pfmdr1*, *Pfcrt*, and the propeller domain of *PfK13* as described elsewhere (*7*). We treated patients with a 3-day regimen of dihydroartemisinin/piperaquine and measured levels of parasitemia on days 0 and 3; this treatment failed in 1 patient with malaria caused by *P. falciparum*. As follow-up for this patient, we collected blood samples from that patient on day 8 to determine piperaquine concentration using liquid chromatographic-tandem mass spectrometry.

We collected adult mosquitoes using human landing catches, CDC light traps, and BG-Sentinel and Suna traps (Biogents, https://www.biogents.com) (Table). We conducted larval prospecting in pools of water in French military camps, Djiboutian military police locations, and the Ambouli Gardens (a public area with a garden market and cattle breeding range). We reared larvae until imago emergence, then identified adult mosquitoes using a morphologic key (Walter Reed Biosystematics Unit, http://vectormap.si.edu/downloads/VHazardReports/VHR_Anopheles_stephensi_2018.pdf). We extracted DNA from the legs of 103 *An. stephensi* mosquitoes and sequenced the cytochrome oxidase C subunit I gene to confirm morphologic identification. In addition, we conducted a phylogenetic analysis (Appendix Figure 2).

**Table:** Adult and larval mosquitoes collected by human landing catches and traps, Djibouti, Republic of Djibouti, 2019*

Species and sampling method†	No. (% female)	Resources/time expended
*Anopheles stephensi*		
HLC	1 (100.0)	2 persons/7 h
BG Sentinel Trap	1 (100.0)	2 traps/120 h
Larval emergence	190 (56.8)	
Subtotal	192 (57.3)	
*Aedes aegypti*		
HLC	11 (100.0)	2 persons/7 h
BG Sentinel Trap	88 (56.8)	2 traps/120 h
Suna Trap	10 (90.0)	2 traps/96 h
CDC Light Trap	2 (100.0)	2 traps/120 h
Larval emergence	32 (46.9)	
Subtotal	143 (60.8)	
*Culex quinquefasciatus*		
HLC	113 (100.0)	2 persons/7 h
BG Sentinel Trap	573 (68.2)	2 traps/120 h
Suna Trap	221 (57.5)	2 traps/96 h
CDC Light Trap	408 (66.2)	2 traps/120 h
Larval emergence	26 (92.3)	
Subtotal	1,341 (69.0)	
Other *Culex* sp.		
HLC	43 (100.0)	2 persons/7 h
BG Sentinel Trap	5 (40.0)	2 traps/120 h
Suna Trap	2 (100.0)	2 traps/96 h
CDC Light Trap	10 (100.0)	2 traps/120 h
Larval emergence	99 (71.7)	
Subtotal	159 (80.5)	
Total	1,835 (68.1)	

*HLC, human landing catch. †Sampling methods were CDC Light traps, BG-Sentinel and Suna traps (Biogents, https://www.biogents.com), as well as HLC and larval emergence in laboratory. Each BG-Sentinel trap was baited with BG-MB5 attractant (Biogents) and a CO_2_ production system (>50,000 ppm) based on the fermentation of sugar, yeast, and agar. For larval emergence method, larvae were collected and reared in laboratory until imago emergence.

**Figure 1:**
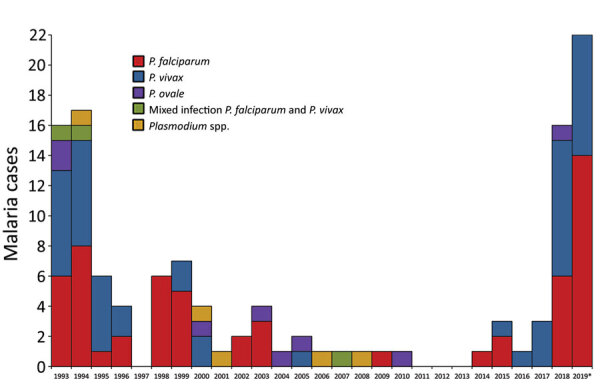
Distribution of 120 malaria cases caused by *Plasmodium* species among French Armed Force members, Djibouti, Republic of Djibouti, 1993–2019. *Data for 2019 include 2 *P. falciparum* infections among service members’ families, 1 *P. vivax* relapse, and 3 *P. vivax* infections in France imported from Djibouti.

In the early 2000s, malaria incidence in the FAF was only 1–4 cases per year; during 2011–2013, no cases were documented (Figure 1). Malaria reemerged in 2014 and reached an incidence of 5.9 cases/1,000 persons in 2018 and 8.1 cases/1,000 persons in 2019. In the 2018–19 season, *P. falciparum* and *P. vivax* cocirculated (*P. falciparum* caused 20/38 [53%] cases, *P. vivax* caused 17/38 [45%] cases, and *P. ovale* caused 1 [2%] case). Among the country’s population, incidence increased from 25.5 cases/1,000 persons in 2018 to 49.8 cases/1,000 persons in 2019 (Appendix Figure 1) (*8*). In 2019, we documented 1 instance of treatment failure in a FAF service member with *P.* *falciparum* infection; this patient had a thin blood smear showing a parasitemia level of 2.0%. After 3 days of treatment with dihydroartemisinin/piperaquine, the patient still had a fever and 2.0% parasitemia level. The piperaquine plasma concentration on day 8 was 77.7 ng/mL, above the therapeutic threshold (38.1 ng/mL [95% CI 25.8–59.3] expected on day 7), confirming good regimen adherence and absorption (*9*). This case met the definition for early treatment failure of an artemisinin derivative according to criteria from the World Health Organization (https://apps.who.int/iris/handle/10665/162441). We sequenced molecular markers of resistance to antimalarial drugs for 9 *P. falciparum* isolates (Appendix Table 3). All isolates had molecular markers associated with resistance to mefloquine. In addition, 89% had resistance markers against chloroquine and pyrimethamine or proguanil. We did not observe any mutations in the K13 propeller region (which sometimes contains mutations associated with artemisinin resistance), including the isolate from the patient in whom treatment failed (*10*). In Africa, failures of artemisinin combined therapy potentially caused by K13 mutations observed in Southeast Asia remain rarely described (*11*).

**Figure 2:**
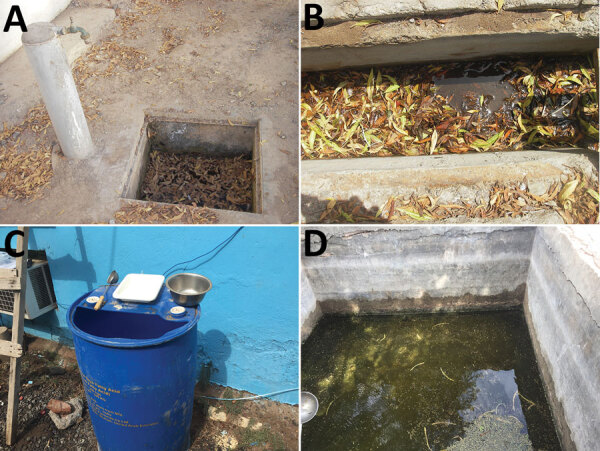
*Anopheles stephensi* breeding sites, Djibouti, Republic of Djibouti, 2019. A) Manhole. B) Ditch. C) Plastic drum. D) Water tank.

We conducted entomologic investigations during a dry period (i.e., February–March). We collected 1,835 adult mosquitoes and larvae: 1,500 *Culex*, 143 *Aedes aegypti*, and 192 *An. stephensi* (Table). We caught 2 adult *An. stephensi* mosquitoes using the human landing catch and BG-Sentinel trap. We identified 25 breeding sites, 15 of which contained *An. stephensi* larvae. All the *An. stephensi* breeding sites were artificial and located in urban or suburban areas; 9/15 also contained *Ae. aegypti* larvae, *Cx. quinquefasciatus* larvae, or both (Appendix Table 2). Examples of *An. stephensi* breeding sites included manholes, ditches, plastic drums, and water tanks (Figure 2). In military camps, standing water was related to leaks and stagnation caused by faulty maintenance of the water distribution and drainage network. The most productive breeding sites (≈800 water tanks with thousands of *An. stephensi* larvae) were near livestock areas, mainly in the Ambouli Gardens district. We confirmed morphologic identification of adult *An. stephensi* mosquitoes by cytochrome oxidase C subunit I sequencing, which identified 8 haplotypes. Phylogenetic trees did not clearly indicate the origin of *An. stephensi* mosquitoes in Djibouti (Appendix Figure 2).

## Conclusions

In the Republic of Djibouti, malaria transmission has increased since 2013. Even populations with strong malaria control programs, such as the FAF, are now affected. In 2018, the country notified the World Health Organization of ≈100,000 suspected cases, mainly among febrile patients with negative results on a rapid diagnostic test (Appendix Figure 1). Considering these suspected cases, we believe the true incidence could be 5 times higher than the 25,319 cases confirmed that year. A recent study (*12*) found a high prevalence (86.5%) of *pf*hrp2 and *pf*hrp3 gene deletion among *P.* *falciparum* parasites in the city of Djibouti. 

We documented an early treatment failure of dihydroartemisinin/piperaquine in an isolate lacking a K13 mutation. This finding could signal the emergence of *P. falciparum* resistance to artemisinin derivatives in Djibouti.

*An. stephensi* mosquitoes are well-established in Djibouti and have been observed in Sudan and Ethiopia (*13*). Our study shows that this species shares breeding sites with *Ae. aegypti* and *Cx. quinquefasciatus* mosquitoes, highlighting its adaptation to urban areas. Models predict broad expansion of *An. stephensi* mosquito distribution into major cities in Africa, where large malaria outbreaks could occur among growing resident populations susceptible to the disease (*14*). Furthermore, a high level of resistance among mosquitoes to all insecticide families (e.g., organochlorates, pyrethroids, carbamates, and organophosphates) has been described in Djibouti and Ethiopia (*8*,*15*). In semiarid regions such as the Republic of Djibouti, residents often store water in plastic drums that act as breeding sites for *An. stephensi* mosquitoes. To control malaria and limit the spread of this anopheline species, communities and governments should prioritize larval control and access to the water distribution network.

Appendix Additional information on role of *Anopheles stephensi* in malaria outbreak, Djibouti, 2019.
