# Supplementary material for: Role of Anopheles stephensi Mosquitoes in Malaria Outbreak, Djibouti, 2019
Source: Emerg Infect Dis. 2021 Jun;27(6):1697–700. doi: 10.3201/eid2706.204557 (PMC8153885; doi:10.3201/eid2706.204557)
Supplement: Appendix — Additional information on role of Anopheles stephensi in malaria outbreak, Djibouti, 2019. [file 20-4557-Techapp-s1.pdf]

Article DOI: https://doi.org/10.3201/eid2706.204557

Role of *Anopheles stephensi* in Malaria Outbreak, Djibouti, 2019

Appendix

Appendix Table 1. Resistance markers of *Plasmodium falciparum* isolates to selected drugs, Djibouti, Republic of Djibouti, 2019*

	Molecular marker (drug of resistance), aa													
	Dihydrofolate reductase (PYR)				Chloroquine resistance transporter (CQ)		Multidrug resistance 1 (MQ)					K13 propeller (artemisinin derivatives)	Resistance profile
Isolate	51	59	108	164	76	356	86	184	1034	1042	1246		
25697	I	R	N	I	CVIET	I	N	F	S	N	D	WT	PYR, CQ, MQ
25700†	I	R	N	I	CVIET	I	N	F	S	N	D	WT	PYR, CQ, MQ
25747	I	R	N	I	CVIET	ND	N	F	S	N	D	WT	PYR, CQ, MQ
25749	I	R	N	I	CVIET	I	N	F	S	N	D	WT	PYR, CQ, MQ
25809	I	R	N	I	CVIET	I	N	F	S	N	D	WT	PYR, CQ, MQ
25810	I	C	N	I	CVIET	I	N	F	S	N	D	WT	PYR, CQ, MQ
25834	I	R	N	I	CVMNK	I	N	F	S	N	D	WT	PYR, MQ
25910	N	C	S	I	CVIET	T	N	F	S	N	D	WT	CQ, MQ
25911	I	R	N	I	CVIET	T	N	F	S	N	D	WT	PYR, CQ, MQ
Drug resistance, no. (%)	8 (88.9)				8 (88.9)		9 (100.0)					0	

*C, cysteine; CQ, chloroquine; D, aspartic acid; E, glutamic acid; F, phenylalanine; I; isoleucine; K; lysine; M, methionine; MQ, mefloquine; N, asparagine; ND, not determined; PYR, pyrimethamine; R, arginine; S, serine; T, threonine; V, valine; WT, wild-type.
†Isolate from patient in whom dihydroartemisinin/piperaquine treatment failed.

Appendix Table 2. Characteristics of *Anopheles stephensi* breeding sites, Djibouti, Republic of Djibouti, 2019*

Location	Setting	Distance from dwellings, m	Type†	Sun exposure	Size, m	Depth, m	Water	Foliage cover	Other mosquito species
BA 188	Urban	<10	Manhole	Partially shaded	<1	<0.5	Clean	Abundant	*Aedes aegypti*
BA 188	Urban	<10	Manhole	Shaded	<1	<0.5	Clean	Abundant	*Ae. aegypti*
BA 188	Urban	10–100	Puddle	Partially shaded	<1	<0.5	Clean	Absent	*Ae. aegypti, Culex quinquefasciatus*
BA 188	Urban	10–100	Puddle	Shaded	1–5	<0.5	Clean	Absent	No
BA 188	Urban	<10	Manhole	Partially shaded	<1	<0.5	Clean	Absent	*Ae. aegypti, Cx. quinquefasciatus*
BA 188	Urban	<10	Ditch	Partially shaded	1–5	<0.5	Clean	Some	No
	Urban	10–100	Water tank	Partially shaded	1–5	<0.5	Clean	Some	*Ae. aegypti, Cx. quinquefasciatus*
RIOM	Urban	10–100	Manhole	Shaded	<1	<0.5	Clean	Some	*Ae. aegypti*
RIOM	Urban	<10	Ditch	Partially shaded	1–5	<0.5	Clean	Abundant	No
RIOM	Urban	10–100	Manhole	Shaded	<1	<0.5	Clean	Absent	No
Naval base héron	Urban	<10	Manhole	Partially shaded	<1	<0.5	Clean	Absent	*Ae. aegypti, Cx. quinquefasciatus*
Djibouti Gendarmerie Nationale brigade, districts 6 and 7	Urban	<10	Plastic drum	Partially shaded	<1	<0.5	Clean	Absent	*Ae. aegypti*
Djibouti Gendarmerie Nationale brigade, Ambouli district	Urban	<10	Water tank	Shaded	<1	<0.5	Clean	Absent	*Ae. aegypti*
Ambouli Gardens	Suburban	10–100	Water tank	Sunny	1–5	<0.5	Clean	Some	*Cx. quinquefasciatus*
Ambouli Gardens	Suburban	10–100	Water tank	Sunny	1–5	<0.5	Clean	Some	*Cx. quinquefasciatus*

*BA, airforce base; RIOM, 5th Interarmées Outre-Mer Regiment.
†(Figure 2).

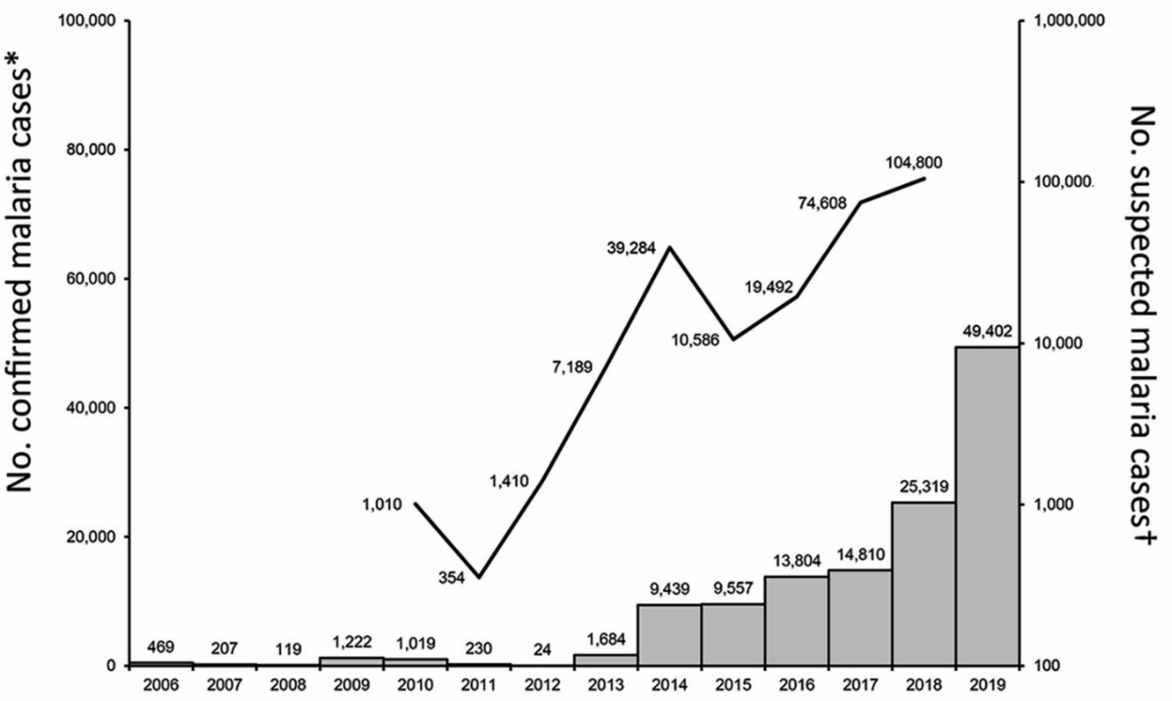

Appendix Figure 1. Distribution of confirmed and suspected malaria among residents, Djibouti, Republic of Djibouti, 2006–2019. Bars indicate confirmed malaria cases (*8*); line indicates suspected cases (*2*).

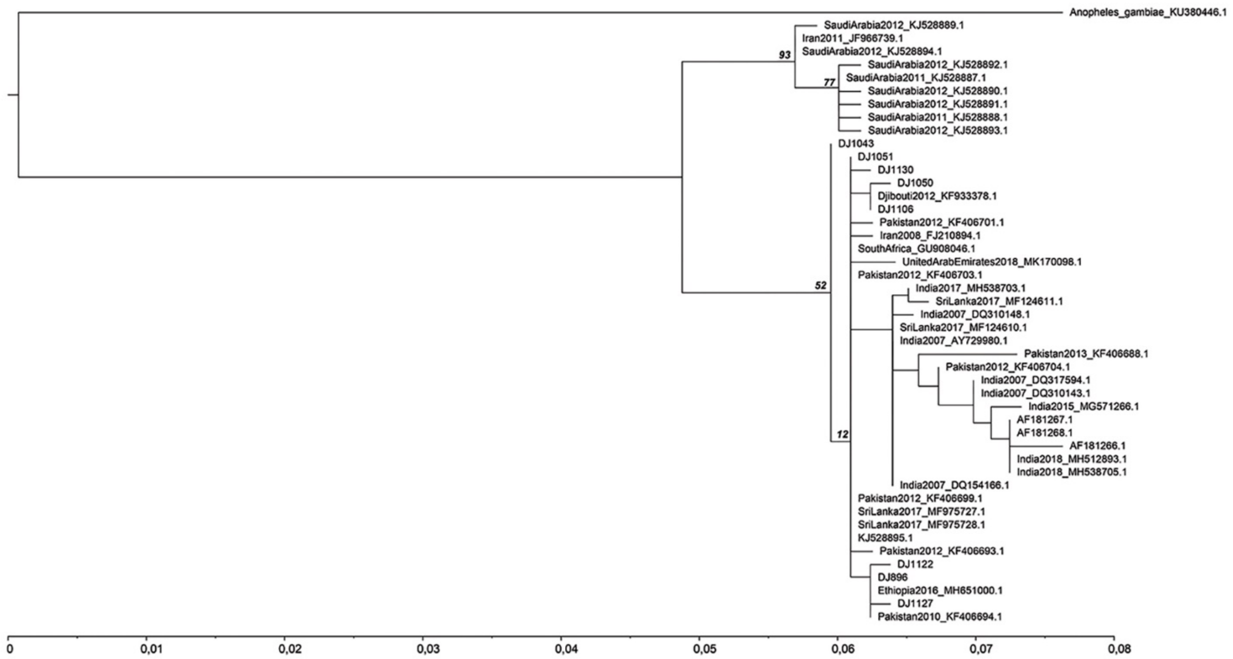

Appendix Figure 2. Phylogenetic tree of cytochrome oxidase C subunit I sequences of *Anopheles stephensi*. Representative haplotypes, analyzed with RAxML version 8.2.10 30 (https://github.com/stamatak/standard-RAxML), generated the maximum-likelihood tree with 100 rapid bootstrap replicates. Topology based on an *Anopheles gambiae* sequence. General time-reversible plus gamma distribution plus invariable site nucleotide substitution model based on corrected Akaike's Information Criterion values according to PartitionFinder version 2 software 31 (https://github.com/brettc/partitionfinder/releases/latest) with the linked branch length option. Phylogenetic trees were visualized using FigTree version 1.4.3 32 (http://tree.bio.ed.ac.uk/software/figtree/). Labels indicate the country and year of sample collection as well as the GenBank accession no. The scale indicates substitutions per nucleotide. The numbers to the left of the main nodes indicate bootstrap values.
